# Tracer experiment revealed that (*E*)-3″-hydroxygeranylhydroquinone is not an intermediate of the shikonin/alkannin and shikonofuran biosynthetic pathways in *Lithospermum erythrorhizon*

**DOI:** 10.5511/plantbiotechnology.24.0303a

**Published:** 2024-09-25

**Authors:** Misaki Manabe, Bunta Watanabe, Haruka Oshikiri, Kojiro Takanashi

**Affiliations:** 1 Department of Science, Graduate School of Science and Technology, Shinshu University, Asahi 3-1-1, Matsumoto, Nagano 390-8621, Japan; 2 Chemistry Laboratory, The Jikei University School of Medicine, Kokuryo 8-3-1, Chofu, Tokyo 182-8570, Japan; 3 Department of Biology, Faculty of Science, Shinshu University, Asahi 3-1-1, Matsumoto, Nagano 390-8621, Japan

**Keywords:** alkannin, 3″-hydroxygeranylhydroquinone, *Lithospermum erythrorhizon*, shikonin, shikonofuran

## Abstract

*Lithospermum erythrorhizon* (Boraginaceae) produces shikonin/alkannin, an enantiomeric pair of red naphthoquinone pigments with diverse biological activities. For the industrial production of shikonin/alkannin derivatives, a cell suspension culture system of *L. erythrorhizon* has been established. To produce shikonin/alkannin derivatives more efficiently in cultured cells, it is essential to understand the shikonin/alkannin biosynthetic pathway, which has not been fully elucidated. A previous study suggested that a conversion of (*Z*)- to (*E*)-3″-hydroxygeranylhydroquinone (3″-OH-GHQ) is a branching point of the shikonin/alkannin biosynthetic pathway and the shikonofuran biosynthetic pathway in *L. erythrorhizon* cell cultures. However, it is not clear whether (*E*)-3″-OH-GHQ is an intermediate of both pathways. This study performed a feeding assay with three deuterium-labeled compounds including (*E*)-3″-OH-GHQ and its (*Z*)-isomer, and showed that (*E*)-3″-OH-GHQ was not involved in the shikonin/alkannin and shikonofuran biosynthetic pathways.

Shikonin and its enantiomeric pair, alkannin, are red naphthoquinone pigments produced in the root bark of several Boraginaceae plants, including *Lithospermum erythrorhizon*. Because shikonin/alkannin derivatives have various biological activities, plants producing these derivatives have been used as crude drugs in traditional medicine in European and Asian countries (Yadav et al. 2022). This property of *L. erythrorhizon* as a crude drug led to the establishment of cell culture systems for the industrial production of shikonin derivatives (Yazaki 2017). These cultured cells have been used to study the shikonin/alkannin biosynthetic pathway (Supplementary Figure S1). The first step of shikonin/alkannin biosynthesis is a coupling reaction of *p*-hydroxybenzoic acid (PHB) and geranyl diphosphate (GPP) to form *m*-geranyl-*p*-hydroxybenzoic acid (GBA) (Yazaki et al. 2002). This intermediate is decarboxylated to geranylhydroquinone (GHQ), which is then hydroxylated at the 3″ position to form (*Z*)-3″-hydroxygeranylhydroquinone ((*Z*)-3″-OH-GHQ) (Wang et al. 2019). (*Z*)-3″-OH-GHQ appeared to be cyclized to deoxyshikonin, followed by hydroxylation and acylation at the side chain to various shikonin/alkannin derivatives (Oshikiri et al. 2020; Song et al. 2021). Although several studies have attempted to identify the enzymes that catalyze the reaction between (*Z*)-3″-OH-GHQ and deoxyshikonin, no enzyme has been identified to date. An enzyme assay using crude cell-free extract of cultured *L. erythrorhizon* cells reported that (*E*)-3″-OH-GHQ is produced from (*Z*)-3″-OH-GHQ (Yamamoto et al. 2020). In *Arnebia euchroma*, a plant of the Boraginaceae family, an enzyme of the cinnamyl alcohol dehydrogenase family (AeHGO) catalyzes this reaction (Wang et al. 2023). It has been suggested that this (*Z*)- to (*E*)-form conversion is a branching point of the two pathways leading to shikonin/alkannin and shikonofuran (Yamamoto et al. 2020), though it is not clear whether (*E*)-3″-OH-GHQ is an intermediate of both pathways and other biosynthetic pathways. Identification of the intermediates and the reaction mechanisms of these biosynthetic pathways is strongly desired to establish more efficient production of shikonin/alkannin derivatives in cultured cells. In this study, a feeding assay with deuterium-labeled (*E*)-3″-OH-GHQ was performed on *L. erythrorhizon* cells to investigate the involvement of this compound in the shikonin/alkannin and shikonofuran biosynthetic pathways.

As substrates for the feeding assay, we synthesized a deuterium-labeled GHQ and (*Z*)-3″-OH-GHQ in addition to (*E*)-3″-OH-GHQ (GHQ-*d*_3_, (*Z*)-3″-OH-GHQ-*d*, (*E*)-3″-OH-GHQ-*d*) (Figure 1A) as described in Supplementary Protocol S1. *L. erythrorhizon* cell cultures, maintained as previously reported (Takanashi et al. 2019), were inoculated into M9 medium (Fujita et al. 1981) and cultured in the dark for 3 days. Subsequently, 200 µM of deuterium-labeled compound in methanol was added to the cells and cultured for another 3 days. The same volume of methanol was added as a control. After the pigment-producing cells were collected by suction filtration, 100 mg of each were ground with metal beads, and extracted with a 10-fold (v/w) of methanol for 1 h at room temperature. The supernatant was filtered and analyzed by HPLC-mass spectrometry using an LCMS-8040 system (Shimadzu) equipped with a COSMOSIL 2.5C18-MS-II column (2.0 mm I.D.×100 mm; Nacalai Tesque) at a flow rate of 0.2 ml min^−1^ at 30°C. The mobile phase consisted of water containing 1% (v/v) formic acid and acetonitrile under the following gradient condition: 80/20 (v/v) at 0 min, 33/67 at 18 min, 0/100 at 19.5–24 min, and 80/20 at 24–30 min. Mass spectra were obtained in both positive and negative modes. To evaluate the effect of labeled compounds on the production of shikonin/alkannin derivatives and shikonofuran derivatives, the uptake of labeled compounds in both pathways was measured.

**Figure 1:**
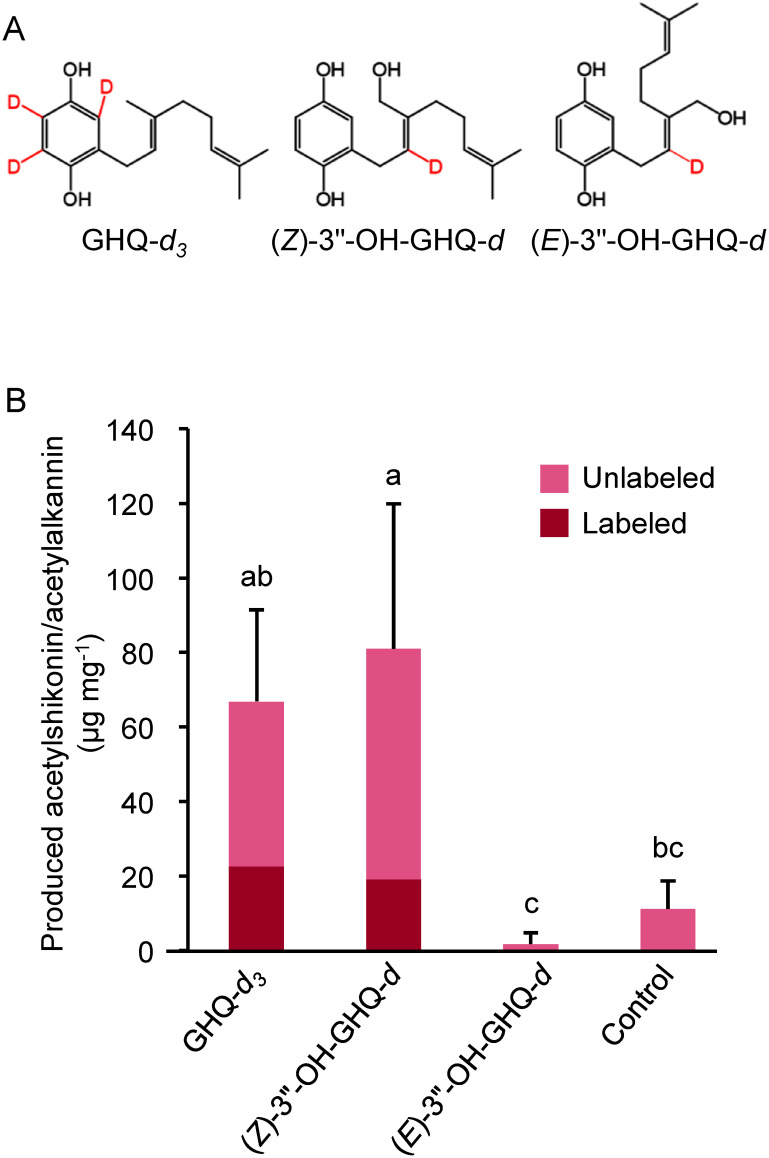
Figure 1. Production of acetylshikonin/acetylalkannin by *L. erythrorhizon* cells treated with labeled compounds. (A) Deuterium-labeled compounds used in this study. The method of synthesis of these compounds is described in Supplementary Protocol S1. (B) Comparison of acetylshikonin/acetylalkannin production and presence of labeled compounds. The labeled compounds were detected when treated with GHQ-*d*_3_ or (*Z*)-3″-OH-GHQ-*d*. Significant differences (*p*<0.05) of means±SD (*n*=3) were determined by Turkey–Kramer test and are indicated by different lowercase letters.

Because cultured *L. erythrorhizon* cells mainly produce acetylated shikonin/alkannin derivatives, we first investigated their production and conversion rates of labeled compounds. The production of acetylshikonin/acetylalkannin was increased when GHQ-*d*_3_ or (*Z*)-3″-OH-GHQ-*d* was added (Figure 1B). The conversion of applied GHQ-*d*_3_ and (*Z*)-3″-OH-GHQ-*d* was observed from the mass shifts of molecular ion of acetylshikonin/acetylalkannin, *m*/*z* 330 to *m*/*z* 331 (Supplementary Figure S2A), and the percentage of the labeled form in the total production was on average 33.9% and 23.4%, respectively (Figure 1B).

In contrast, acetylshikonin/acetylalkannin production in (*E*)-3″-OH-GHQ-*d* treated cells was smaller than that in the GHQ-*d*_3_ and (*Z*)-3″-OH-GHQ-*d* treated cells, and no labeled acetylshikonin/acetylalkannin was detected (Supplementary Figure S2A), indicating that (*E*)-3″-OH-GHQ is not a precursor of acetylshikonin/acetylalkannin. Next, we measured the conversion rates of the labeled compounds to deoxyshikonofuran, a product of the shikonofuran biosynthetic pathway (Yazaki et al. 1986). The labeled deoxyshikonofuran was observed when GHQ-*d*_3_ or (*Z*)-3″-OH-GHQ-*d* was applied with the mass shift of the molecular ion, *m*/*z* 259 to *m*/*z* 262 or *m*/*z* 260, respectively (Supplementary Figure S2B). The conversion rates of GHQ-*d*_3_ and (*Z*)-3″-OH-GHQ-*d* were on average 32.0% and 33.3%, respectively (Figure 2). When (*E*)-3″-OH-GHQ-*d* was added, deoxyshikonofuran tended to decrease and no labeled form was detected (Figure 2, Supplementary Figure S2B), similar to the result for acetylshikonin/acetylalkannin.

**Figure 2:**
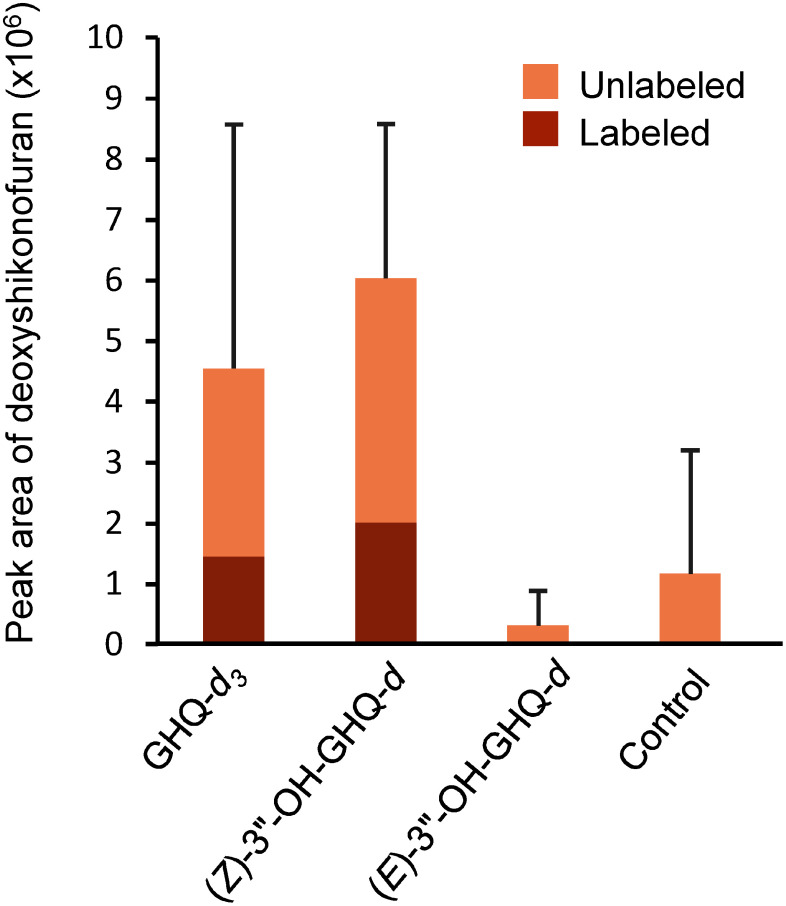
Figure 2. Production of deoxyshikonofuran. The presence of labeled compounds was shown. The labeled compounds were detected when only GHQ-*d*_3_ or (*Z*)-3″-OH-GHQ-*d* was treated. Significant differences (*p*<0.05) of means±SD (*n*=3) were calculated by Turkey–Kramer test and no significant differences were detected.

This study clearly demonstrated that (*E*)-3″-OH-GHQ-*d* is not an intermediate of shikonin/alkannin and shikonofuran biosynthetic pathways, since no applied deuterium-labeled compound was converted to them (Figure 3). In contrast, detection of labeled acetylshikonin/acetylalkannin and deoxyshikonofuran indicated that GHQ and (*Z*)-3″-OH-GHQ are the biosynthetic intermediates of these products (Figure 3). To increase the production of shikonin/alkannin derivatives in cultured cells, the addition of GHQ and/or (*Z*)-3″-OH-GHQ would be efficient, especially GHQ can be easily synthesized from inexpensive hydroquinone and geraniol via single chemical reaction (Baeza et al. 2012). Characterization of the enzymes that catalyze the cyclization of (*Z*)-3″-OH-GHQ to deoxyshikonin would also be effective.

**Figure 3:**
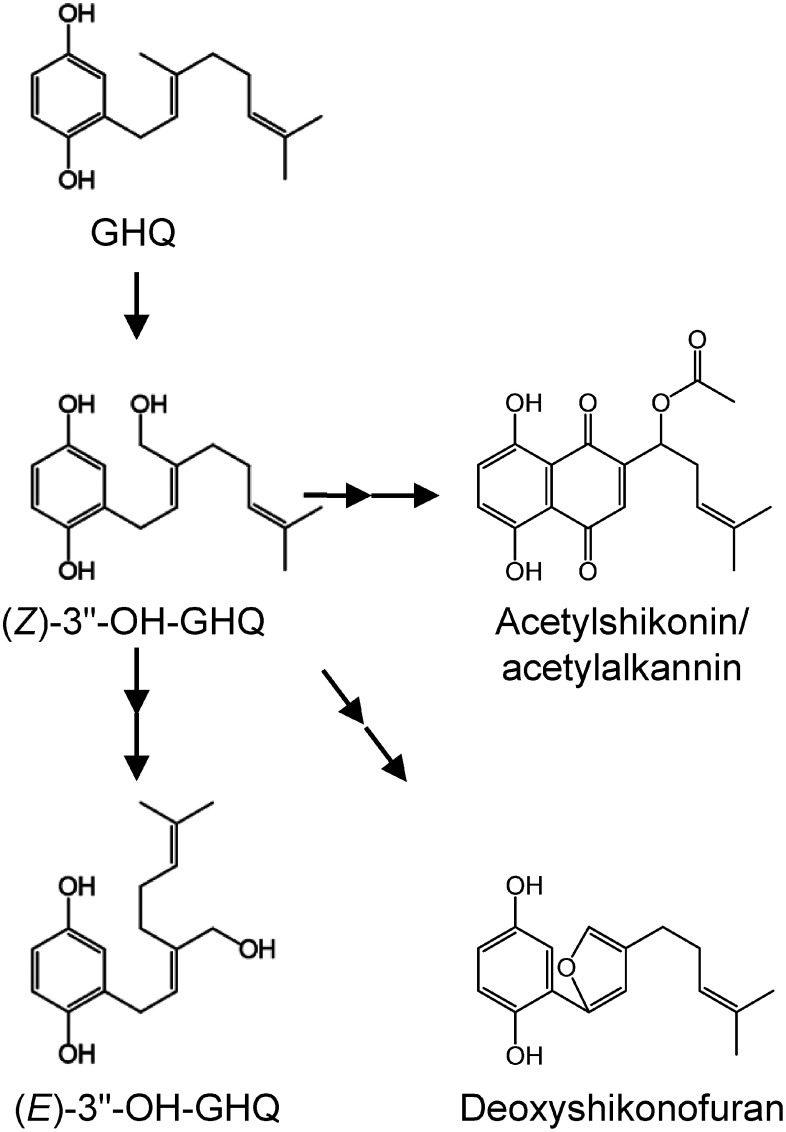
Figure 3. Biosynthetic pathway proposed by this study. (*E*)-3″-OH-GHQ is not an intermediate of both shikonin/alkannin and shikonofuran biosynthetic pathways.

## Abbreviations

GBA: *m*-geranyl-*p*-hydroxybenzoic acid
GHQ: geranylhydroquinone
GPP: geranyl diphosphate
PHB: *p*-hydroxybenzoic acid
3″-OH-GHQ: 3″-hydroxygeranylhydroquinone

## Description of Supplementary Files

Supplementary Figure S1. Proposed biosynthetic pathways of shikonin/alkannin and shikonofuran derivatives before this study.

Supplementary Figure S2. Mass spectra of products treated with labeled compounds.

Supplementary Protocol S1. Synthesis of deuterium-labeled GHQ, (*Z*)-3″-OH-GHQ, and (*E*)-3″-OH-GHQ.

## References

[RBaeza2012] Baeza E, Catalán K, Peña-Cortés H, Espinoza L, Villena J, Carrasco H (2012) Synthesis of geranylhydroquinone derivatives with potencial cytotoxic activity. *Quim Nova* 35: 523–526

[RFujita1981] Fujita Y, Hara Y, Suga C, Morimoto T (1981) Production of shikonin derivatives by cell suspension cultures of *Lithospermum erythrorhizon*: II. A new Medium for the production of shikonin derivatives. *Plant Cell Rep* 1: 61–63 24258860 10.1007/BF00269273

[ROshikiri2020] Oshikiri H, Watanabe B, Yamamoto H, Yazaki K, Takanashi K (2020) Two BAHD acyltransferases catalyze the last step in the shikonin/alkannin biosynthetic pathway. *Plant Physiol* 184: 753–761 32727911 10.1104/pp.20.00207 PMC7536692

[RSong2021] Song W, Zhuang Y, Liu T (2021) CYP82AR subfamily proteins catalyze C-1′ hydroxylations of deoxyshikonin in the biosynthesis of shikonin and alkannin. *Org Lett* 23: 2455–2459 33728922 10.1021/acs.orglett.1c00360

[RTakanashi2019] Takanashi K, Nakagawa Y, Aburaya S, Kaminade K, Aoki W, Saida-Munakata Y, Sugiyama A, Ueda M, Yazaki K (2019) Comparative proteomic analysis of *Lithospermum erythrorhizon* reveals regulation of a variety of metabolic enzymes leading to comprehensive understanding of the shikonin biosynthetic pathway. *Plant Cell Physiol* 60: 19–28 30169873 10.1093/pcp/pcy183

[RWang2023] Wang R, Liu C, Lyu C, Sun J, Kang C, Ma Y, Wan X, Guo J, Shi L, Wang J, et al. (2023) The discovery and characterization of AeHGO in the branching route from shikonin biosynthesis to shikonofuran in *Arnebia euchroma.* *Front Plant Sci* 14: 1160571 37180378 10.3389/fpls.2023.1160571 PMC10167036

[RWang2019] Wang S, Wang R, Liu T, Lv C, Liang J, Kang C, Zhou L, Guo J, Cui G, Zhang Y, et al. (2019) CYP76B74 catalyzes the 3″-hydroxylation of geranylhydroquinone in shikonin biosynthesis. *Plant Physiol* 179: 402–414 30498024 10.1104/pp.18.01056 PMC6426415

[RYadav2022] Yadav S, Sharma A, Nayik GA, Cooper R, Bhardwaj G, Sohal HS, Mutreja V, Kaur R, Areche FO, AlOudat M, et al. (2022) Review of shikonin and derivatives: Isolation, chemistry, biosynthesis, pharmacology and toxicology. *Front Pharmacol* 13: 905755 35847041 10.3389/fphar.2022.905755 PMC9283906

[RYamamoto2020] Yamamoto H, Tsukahara M, Yamano Y, Wada A, Yazaki K (2020) Alcohol dehydrogenase activity converts 3″-hydroxy-geranylhydroquinone to an aldehyde intermediate for shikonin and benzoquinone derivatives in *Lithospermum erythrorhizon.* *Plant Cell Physiol* 61: 1798–1806 32810231 10.1093/pcp/pcaa108

[RYazaki2017] Yazaki K (2017) *Lithospermum erythrorhizon* cell cultures: Present and future aspects. *Plant Biotechnol (Tokyo)* 34: 131–142 31275019 10.5511/plantbiotechnology.17.0823a PMC6565996

[RYazaki1986] Yazaki K, Fukui H, Tabata M (1986) Isolation of the intermediates and related metabolites of shikonin biosynthesis from *Lithospermum erythrorhizon.* *Chem Pharm Bull* 34: 2290–2293

[RYazaki2002] Yazaki K, Kunihisa M, Fujisaki T, Sato F (2002) Geranyl diphosphate: 4-hydroxybenzoate geranyltransferase from *Lithospermum erythrorhizon*: Cloning and characterization of a key enzyme in shikonin biosynthesis. *J Biol Chem* 277: 6240–6246 11744717 10.1074/jbc.M106387200

