# Supplementary material for: Tracer experiment revealed that (E)-3″-hydroxygeranylhydroquinone is not an intermediate of the shikonin/alkannin and shikonofuran biosynthetic pathways in Lithospermum erythrorhizon
Source: Plant Biotechnol (Tokyo). 2024 Sep 25;41(3):315–7. doi: 10.5511/plantbiotechnology.24.0303a (PMC11921126; doi:10.5511/plantbiotechnology.24.0303a)
Supplement: Supplementary Data [file plantbiotechnology-41-3-24.0303a-s001.pdf]

Supplementary File

Tracer experiment revealed that (*E*)-3"-hydroxygeranylhydroquinone is not an intermediate of the shikonin/alkannin and shikonofuran biosynthetic pathways in *Lithospermum erythrorhizon*

Misaki Manabe, Bunta Watanabe, Haruka Oshikiri, Kojiro Takanashi

Supplementary Figure S1-2
Supplementary Protocol S1

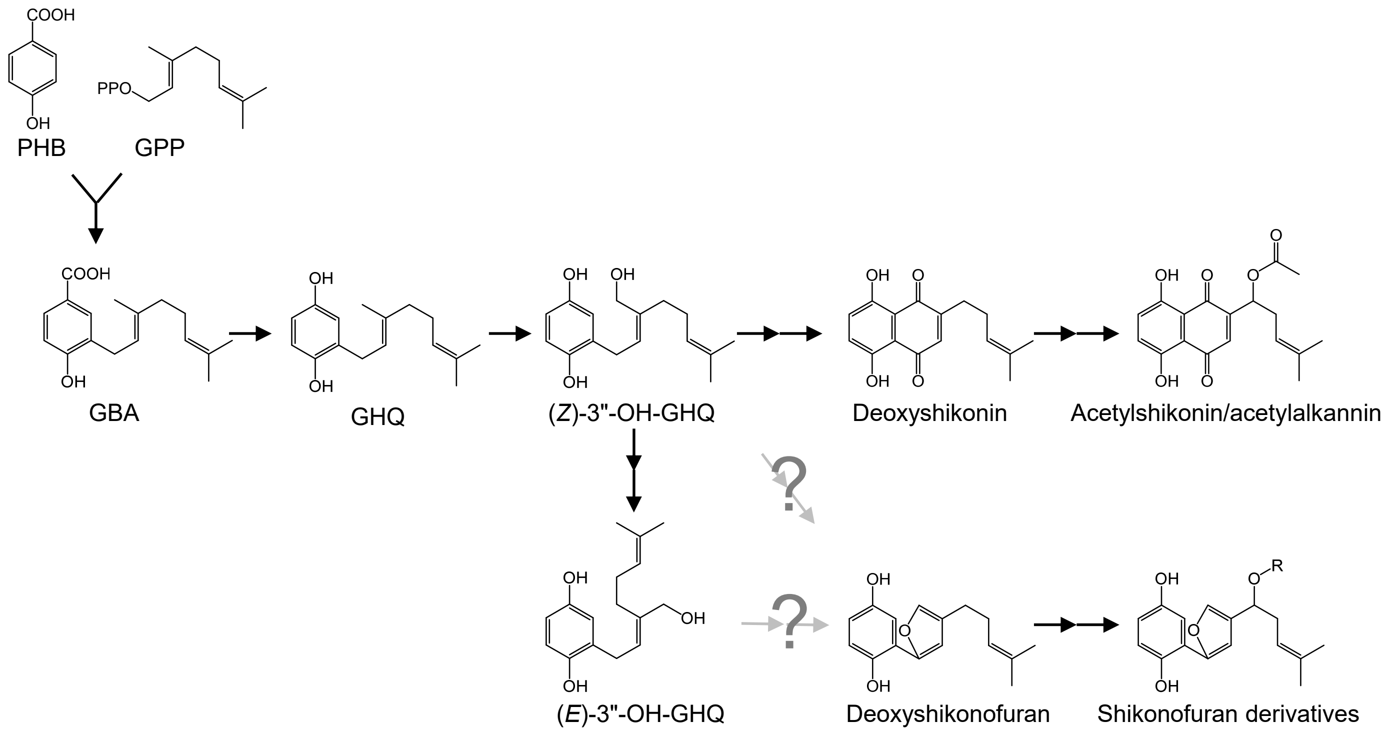

Supplementary Figure S1. Proposed biosynthetic pathways of shikonin/alkannin and shikonofuran derivatives before this study.

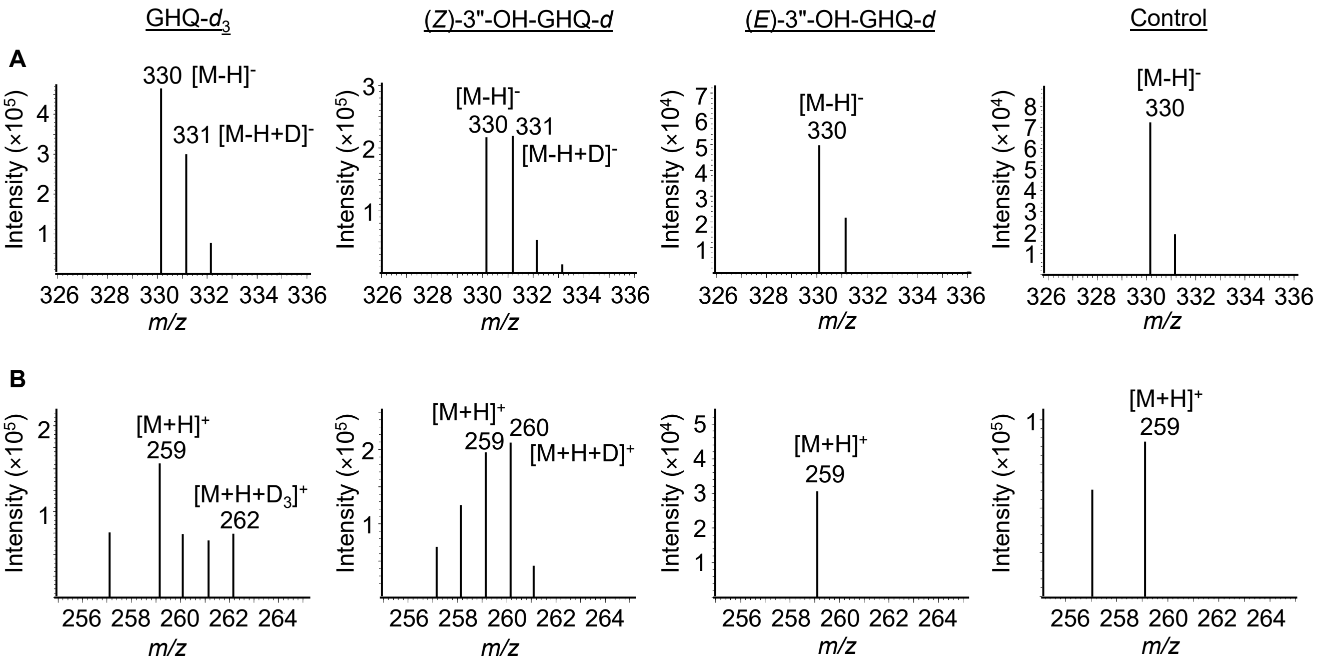

Supplementary Figure S2. Mass spectra of products treated with labeled compounds. Mass spectra of (A) acetylshikonin/acetylalkannin (negative mode) and (B) deoxyshikonofuran (positive mode). Labeled molecular ions were detected when treated with GHQ-d_3 or (*Z*)-3"-OH-GHQ-*d*. M is the molecular weight of the non-labeled molecule.

Supplementary Protocol S1

Synthesis of deuterium-labeled GHQ, (*Z*)-3″-OH-GHQ, and (*E*)-3″-OH-GHQ[§]

General

Melting points (mp) were measured using an AS ONE ATM-01 melting point apparatus and are uncorrected. NMR spectra were obtained using a JEOL JNM-ECA600 spectrometer (600 MHz for ^{1}H; 151 MHz for ^{13}C). Chemical shifts are reported in parts per million relative to the internal standards [tetramethylsilane (0.00 ppm) for ^{1}H; $CDCl_3$ (77.00 ppm) and CD_3OD (49.00 ppm) for ^{13}C]. High-resolution mass spectra were recorded using a Bruker timsTOF spectrometer. Flash column chromatography on silica gel was carried out using a Biotage Isolera One chromatograph with SNAP Ultra cartridges (silica gel, 25 µm).

Reagents were used as received from common commercial suppliers. **S1** (Baeza et al. 2012), **S3** (Oshikiri et al. 2020), and **S6** (Oshikiri et al. 2020) were prepared according to literature procedures. A pressure tube (Ace pressure tube equipped with front seal plug, Z181099) was purchased from Sigma-Aldrich, Co. (MO, USA).

Synthesis of GHQ-3,4,6-d_3 (S2)

The hydrogen–deuterium exchange reaction at *ortho* and *para* positions of phenolic hydroxy groups developed by us (Watanabe et al. 2021) was adopted (Scheme S1).

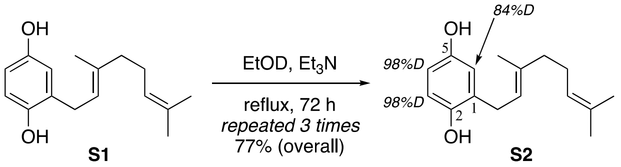

Scheme S1. Synthesis of **S2**.

(*E*)-2-(3,7-Dimethylocta-2,6-dien-1-yl)benzene-3,5,6-d_3-1,4-diol (S2). *The first cycle:* GHQ (**S1**, *E*/*Z* = 87/13, 165 mg, 0.670 mmol) was transferred to a pressure tube, and the inside of the tube was flushed with argon. After the addition of EtOD (8.0 ml, 0.14 mol) and Et_3N (0.30 ml, 2.1 mmol) into the tube, the upper space was flushed with argon again. The tube was plugged and heated at 100 ºC for 72 h with stirring. The mixture was cooled to room temperature and concentrated in vacuo. The residue was purified by flash column chromatography on silica gel (hexane/EtOAc = 75/25→50/50, v/v) to give a pale brown oil (159 mg). Based on ^{1}H NMR analysis, the deuterium incorporated ratio at 3, 4, and 6 positions of the product was estimated to be 86%, 94%, and 54%, respectively. *The second cycle:* A mixture of the first cycle product (159 mg), EtOD (7.5 ml, 0.13 mol), and Et_3N (0.28 ml, 2.0 mmol) was heated at 100 ºC for 72 h as described above. After cooling to room temperature, the mixture was concentrated in vacuo, and purified by flash column chromatography on silica gel (hexane/EtOAc = 75/25→50/50, v/v) to give a brown oil (151 mg). Based on ^{1}H NMR analysis, the deuterium incorporated ratio at 3, 4, and 6 positions of the product was estimated to be 96%, 98%, and 73%, respectively. *The third cycle:* A mixture of the second cycle product (151 mg), EtOD (7.0 ml, 0.12 mmol), and Et_3N (0.27 ml, 1.9 mmol) was heated at 100 ºC for 72 h. After cooling to room temperature, the mixture was concentrated in vacuo, and purified by flash column chromatography on silica gel (hexane/EtOAc = 80/20→50/50, v/v) to give **S2** (128 mg, 77% for 3 cycles) as a brown oil. Based on

[§]Abbreviations: DIBAL, diisobutylaluminum hydride; DMF, *N*,*N*-dimethylformamide; DMP, Dess-Martin periodinane; GHQ, geranylhydroquinone; mp, melting points; rt, room temperature, TBAF, tetrabutylammonium fluoride; TBS, *tert*-butyldimethylsilyl; THF, tetrahydrofuran.

^{1}H NMR analysis, the deuterium incorporated ratio at 3, 4, and 6 positions of the product was estimated to be 98%, 98%, and 84%, respectively (Scheme S1). The ^{1}H NMR spectrum of the *E*-isomer was following (600 MHz, $CDCl_3$, δ): 1.60 (3H, s), 1.69 (3H, s), 1.75 (3H, s), 2.06–2.14 (4H, m), 3.30 (2H, d, J = 6.9 Hz), 4.42 (1H, br s), 4.75 (1H, br s), 5.05–5.09 (1H, m), 5.27–5.31 (1H, m), 6.58 (0.02H, br s), 6.61 (0.16H, br s), 6.68 (0.02H, br s). LC–MS m/z (% relative intensity, ion): 251 (19.6, M + 2), 250 (100, M + 1), 249 (68.7, M+), 248 (73.4, M – 1); M = C16H19D3O2.

Synthesis of (*Z*)-3″-OH-GHQ-2′-*d* (S8) and its (*E*)-isomer (S9)

The synthesis of non-labeled (*Z*)- and (*E*)-3″-OH-GHQ reported by us (Oshikiri et al. 2020) was adopted (Scheme S2). The crude product of the $LiAlD_4$ reduction of **S3** was treated with TBSCl, since one of the TBS groups on the phenolic hydroxy groups, probably at the 2-position of the benzene ring, seemed to be removed during the reduction. Selective removal of TBS groups on primary alkyl hydroxy groups over phenolic hydroxy groups (Lane et al. 2005, Dong et al. 2011) was then conducted, since the re-protection of the phenolic hydroxy group also etherified the alkyl hydroxy group of **S4**. Similar to the previous report (Oshikiri et al. 2020), dihydrofuran **S10** was concomitantly obtained during the removal of the TBS groups of **S7**.

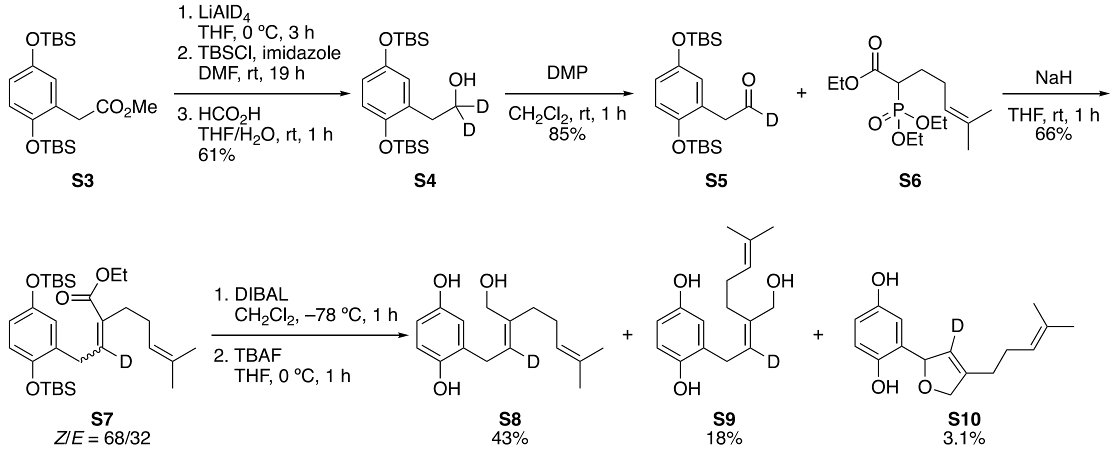

Scheme S2. Synthesis of **S8** and **S9**.

2-{2,5-Bis(*tert*-butyldimethylsiloxy)phenyl}ethan-1,1-*d*₂-1-ol (S4). $LiAlD_4$ (239 mg, 5.69 mmol) was added portionwise to a solution of **S3** (1.02 g, 2.48 mmol) in anhydrous THF (25 ml) at 0 ºC. After stirring for 3 h at 0 ºC, the reaction was quenched by the dropwise addition of water (0.25 ml). An aqueous NaOH solution (10% w/v, 0.25 ml) and water (0.75 ml) was added successively, and the resultant mixture was stirred at 0 ºC for 30 min then at room temperature for 1 h. The mixture was diluted with EtOAc (25 ml) and dried over anhydrous $MgSO_4$. The insoluble materials were filtered off, and the filtrated was concentrated in vacuo to give colorless solid (580 mg). The product and TBSCl (944 mg, 6.26 mmol) was dissolved in anhydrous DMF (5.0 ml). After addition of imidazole (503 mg, 7.39 mmol) at 0 ºC, the mixture was stirred for 19 h at room temperature. The mixture was diluted with water (25 ml), and extracted with toluene (1 × 30 ml, 2 × 10 ml). The combined organic layers were washed with brine (15 ml), dried over anhydrous $MgSO_4$ and filtered. The filtrated was concentrated in vacuo to give colorless oil (1.03 g). This material was dissolved in a mixture of THF (24 ml) and water (4.0 ml). Formic acid (12 ml) was added to the solution, and the reaction mixture was stirred for 1 h at room temperature before pouring onto water (50 ml). The aqueous layer was extracted with toluene (3 × 30 ml), and the combined organic layer was washed successively with water (30 ml), a saturated aqueous solution of $NaHCO_3$ (30 ml), and brine (30 ml). The organic layer was dried over anhydrous $MgSO_4$ and filtered, concentrated in vacuo, and purified by flash column chromatography on silica gel (hexane/EtOAc = 95/5→85/15, v/v) to give **S4** (581 mg, 73% for 3 steps) as a colorless

oil, which solidified after standing for 2 weeks at room temperature. Mp 46 ºC. ^{1}H NMR (600 MHz, $CDCl_3$, δ): 0.16 (6H, s), 0.21 (6H, s), 0.97 (9H, s), 1.00 (9H, s), 2.79 (2H, s), 6.58 (1H, dd, J = 8.6, 2.9 Hz), 6.655 (1H, d, J = 2.9 Hz), 6.657 (1H, d, J = 8.6 Hz). ^{13}C NMR (151 MHz, $CDCl_3$, δ): –4.50 (2C), –4.21 (2C), 18.12, 18.17, 25.68 (3C), 25.78 (3C), 34.10, 62.08 (quint, J = 22.0 Hz), 118.35, 118.95, 122.33, 129.74, 147.98, 149.43. HRMS-ESI (*m*/*z*): $[M+Na]^+$ calcd for $C_{20}H_{36}D_2O_3Si_2Na$, 407.2377; found, 407.2373.

2-{2,5-Bis(*tert*-butyldimethylsiloxy)phenyl}acetaldehyde-1-*d* (S5). Dess-Martin periodinane (754 mg, 1.78 mmol) was added to a solution of **S4** (568 mg, 1.48 mmol) in anhydrous CH_2Cl_2 (7.5 ml) at 0 ºC, and the mixture was stirred for 1 h at room temperature. The reaction mixture was diluted with $CHCl_3$ (40 ml), and washed with a saturated aqueous solution of $NaHCO_3$ containing $Na_2S_2O_3•5H_2O$ (20%, w/v). The separated organic layer was dried over anhydrous $MgSO_4$ and filtered, concentrated in vacuo, and purified by flash column chromatography on silica gel (hexane/EtOAc = 95/5→90/10, v/v) to give **S5** (480 mg, 85%) as a colorless oil. ^{1}H NMR (600 MHz, $CDCl_3$, δ): 0.17 (6H, s), 0.21 (6H, s), 0.97 (9H, s), 0.98 (9H, s), 3.55 (2H, s), 6.63 (1H, d, J = 2.7 Hz), 6.65 (1H, dd, J = 8.6, 2.7 Hz), 6.72 (1H, d, J = 8.6 Hz). ^{13}C NMR (151 MHz, $CDCl_3$, δ): –4.52 (2C), –4.21 (2C), 18.11, 18.16, 25.65 (3C), 25.74 (3C), 45.43 (t, J = 3.6 Hz), 118.85, 119.53, 122.63, 123.82, 148.24, 149.56, 199.73 (t, J = 26.7 Hz). HRMS-ESI (*m*/*z*): $[M+H]^+$ calcd for $C_{20}H_{36}DO_3Si_2$, 382.2339; found, 382.2335.

Ethyl 2-{2-[2,5-bis(*tert*-butyldimethylsiloxy)phenyl]ethylidene-1-*d*}-6-methylhept-5-enoate (S7). NaH (60% in oil, 58.0 mg, 1.45 mmol) was added to a solution of **S6** (472 mg, 1.54 mmol) in anhydrous THF (10 ml) at room temperature, and the mixture was stirred for 20 min under reflux. After cooling to room temperature, a solution of **S5** (465 mg, 1.22 mmol) in anhydrous THF (5.0 ml) was added dropwise to the mixture, and the reaction mixture was stirred for 1 h at room temperature. Silica gel (5 g) was added to the mixture, and the solvent was removed in vacuo. The residue was subjected to flash column chromatography on silica gel (hexane/EtOAc = 99/1→95/5, v/v) to give **S7** (429 mg, 85%, *Z*/*E* = 68/32) as a yellow oil. ^{1}H NMR (600 MHz, $CDCl_3$, δ): 0.15 (1.92H, s), 0.16 (4.08H, s), 0.19 (4.08H, s), 0.21 (1.92H, s), 0.959 (2.88H, s), 0.961 (6.12H, s), 0.99 (6.12H, s), 1.00 (2.88H, s), 1.28 (0.96H, t, J = 7.1 Hz), 1.32 (2.04H, t, J = 7.1 Hz), 1.57 (2.04H, br s), 1.60 (0.96H, br s), 1.66 (2.04H, br d, J = 0.7 Hz), 1.68 (0.96H, br s), 2.09–2.15 (2H, m), 2.28 (1.36H, dd, J = 8.2, 7.2 Hz), 2.41 (0.64H, dd, J = 7.9, 7.6 Hz), 3.43 (0.64H, s), 3.69 (1.36H, s), 4.19 (0.64H, q, J = 7.1 Hz), 4.24 (1.36H, q, J = 7.1 Hz), 5.08–5.12 (0.68H, m), 5.15–5.18 (0.32H, m), 6.55 (0.68H, dd, J = 8.6, 3.1 Hz), 6.56–6.58 (0.64H, m), 6.64 (0.68H, d, J = 8.6 Hz), 6.65 (0.68H, d, J = 3.1 Hz), 6.65–6.66 (0.32H, m). ^{13}C NMR (151 MHz, $CDCl_3$, δ): –4.50 (2C), –4.23 (1.36C), –4.18 (0.64C), 14.26 (0.32C), 14.29 (0.68C), 17.60 (1C), 18.13 (1C), 18.16 (0.68C), 18.21 (0.32C), 25.65 (0.68C), 25.70 (3.32C), 25.77 (2.04C), 25.78 (0.96C), 26.98 (0.32C), 27.67 (0.32C), 27.85 (0.68C), 29.30 (0.32C), 30.53 (0.68C), 34.66 (0.68C), 60.04 (0.68C), 60.24 (0.32C), 117.98 (0.68C), 118.13 (0.32C), 118.77 (0.32C), 118.85 (0.68C), 121.20 (0.32C), 121.51 (0.68C), 123.51 (0.68C), 123.61 (0.32C), 130.33 (0.32C), 131.65 (0.68C), 131.89 (0.68C), 132.08 (0.68C), 132.20 (0.32C), 132.48 (0.32C), 139.72 (0.68C, t, J = 23.1 Hz), 140.15 (0.32C, t, J = 24.6 Hz), 147.52 (0.68C), 147.56 (0.32C), 149.46 (1C), 167.76 (0.32C), 168.00 (0.68C). HRMS-ESI (*m*/*z*): $[M+Na]^+$ calcd for $C_{30}H_{51}DO_4Si_2Na$, 556.3359; found, 556.3352.

(*Z*)-2-[(2,5-Dihydroxyphenyl)ethylidene-1-*d*]-6-methylhept-5-en-1-ol (S8) and its (*E*)-isomer (S9), and 2-[4-(4-methylpent-3-en-1-yl)-2,5-dihydrofuran-2-yl-3-*d*]benzene-1,4-diol (S10). DIBAL (1.0 M in toluene, 2.0 ml, 2.0 mmol) was added to a solution of **S7** (416 mg, 0.779 mmol) in anhydrous CH_2Cl_2 (10 ml) at –78 ºC under argon atmosphere, and the mixture was stirred for 1 h at –78ºC. The reaction was quenched by the dropwise addition of water (0.30 ml) at –78 ºC. The mixture was stirred at room temperature, diluted with $CHCl_3$ (15 ml), dried over anhydrous $MgSO_4$ and filtered, and concentrated in vacuo to give a pale yellow oil (367 mg). This material was

dissolved in anhydrous THF (10 ml), and TBAF (1 M in THF, 3.0 ml, 3 mmol) was added dropwise to the mixture at 0 ºC under argon atmosphere. The reaction mixture was stirred for 1 h at 0 ºC and concentrated in vacuo. The residue was purified by flash column chromatography on silica gel (hexane/EtOAc = 60/40→30/70, v/v) to **S8** (88.7 mg, 43% for 2 steps) as a brown oil. In addition, **S9** (36.6 mg, 18%, brown oil) and **S10** (6.4 mg, 3.3%, pale yellow oil) was also obtained. **S10** was eluted first followed by **S8**, and **S9** was eluted last. **S8**: ^{1}H NMR (600 MHz, CD_3OD, δ): 1.58 (3H, s), 1.64 (3H, s), 2.11–2.17 (4H, m), 3.33 (2H, s), 4.20 (2H, s), 5.09–5.13 (1H, m), 6.47 (1H, dd, *J* = 8.6, 3.0 Hz), 6.55 (1H, d, *J* = 3.0 Hz), 6.59 (1H, d, *J* = 8.6 Hz). ^{13}C NMR (151 MHz, CD_3OD, δ): 17.76, 25.85, 27.85, 29.07, 36.21, 60.21, 114.17, 116.66, 117.36, 125.32, 127.12 (t, *J* = 23.1 Hz), 129.50, 132.30, 139.59, 148.83, 151.13. HRMS-ESI (*m*/*z*): [M+Na]$^+$ calcd for $C_{16}H_{21}DO_3Na$, 286.1524; found, 286.1526. **S9**: ^{1}H NMR (600 MHz, CD_3OD, δ): 1.61 (3H, s), 1.66 (3H, s), 2.11–2.15, 2H, m), 2.20–2.23 (2H, m), 3.31 (2H, s), 4.01 (2H, s), 5.14–5.19 (1H, m), 6.45 (1H, dd, *J* = 8.5, 2.9 Hz), 6.55 (1H, d, *J* = 2.9 Hz), 6.59 (1H, d, *J* = 8.5 Hz), ^{13}C NMR (151 MHz, CD_3OD, δ): 17.74, 25.89, 28.08, 28.71, 28.90, 67.07, 114.01, 116.50, 117.25, 125.36, 125.45 (t, *J* = 22.4 Hz), 129.75, 132.56, 140.40, 148.97, 151.09. HRMS-ESI (*m*/*z*): [M+Na]$^+$ calcd for $C_{16}H_{21}DO_3Na$, 286.1524; found, 286.1524. **S10**: ^{1}H NMR (600 MHz, $CDCl_3$, δ): 1.61 (3H, s), 1.69 (3H, s), 2.13–2.21 (4H, m), 4.65 (1H, dd, *J* = 12.2, 4.0 Hz), 4.70 (1H, dd, *J* = 12.2, 5.3), 5.08–5.12 (1H, m), 5.98–6.01 (1H, m), 6.52 (1H, d, *J* = 3.1 Hz), 6.63 (1H, dd, *J* = 8.6, 3.1 Hz), 6.72 (1H, d, *J* = 8.6 Hz), 7.50 (1H, br s). ^{1}H NMR (600 MHz, CD_3OD, δ): 1.61 (3H, s), 1.67 (3H, s), 2.15–2.23 (4H, m), 4.60 (1H, dd, *J* = 12.0, 3.4 Hz), 4.71 (1H, dd, *J* = 12.0, 5.5 Hz), 5.12–5.16 (1H, m), 6.01–6.03 (1H, m), 6.51 (1H, dd, *J* = 8.6, 3.1 Hz), 6.59 (1H, d, *J* = 8.6 Hz), 6.67 (1H, d, *J* = 3.1 Hz). ^{13}C NMR (151 MHz, CD_3OD, δ): 17.78, 25.84, 27.40, 28.10, 77.88, 84.52, 114.22, 115.66, 116.79, 123.58 (t, *J* = 25.3 Hz), 124.84, 130.93, 133.13, 140.81, 148.14, 151.30. HRMS-ESI (*m*/*z*): [M+Na]$^+$ calcd for $C_{16}H_{19}DO_3Na$, 284.1367; found, 284.1371.

References

Baeza E, Catalán K, Peña-Cortés H, Espinoza L, Villena J, Carrasco H (2012) Synthesis of geranylhydroquinone derivatives with potencial cytotoxic activity. *Quim Nova* 35: 523–526

Dong W, Liu W, Liao X, Guan B, Chen S, Liu Z (2011) Asymmetric total synthesis of (−)-saframycin A from L-tyrosine. *J Org Chem* 76: 5363–5368

Lane JW, Chen Y, Williams RM (2005) Asymmetric total syntheses of (−)-jorumycin, (−)-renieramycin G, 3-epi-jorumycin, and 3-epi-renieramycin G. *J Am Chem Soc* 127: 12684–12690

Oshikiri H, Watanabe B, Yamamoto H, Yazaki K, Takanashi K (2020) Two BAHD Acyltransferases catalyze the last step in the shikonin/alkannin biosynthetic pathway *Plant Physiol* 184: 753–761

Watanabe B, Nishitani S, Koeduka T (2021) Synthesis of deuterium-labeled cinnamic acids: Understanding the volatile benzenoid pathway in the flowers of the Japanese loquat *Eriobotrya japonica*. *J Labelled Compd Radiopharm* 64: 403–416
